# Editorial: Community series: expanding the science of compassion, volume II

**DOI:** 10.3389/fpsyg.2023.1341792

**Published:** 2024-01-05

**Authors:** Myriam Mongrain, James Kirby, Dacher Keltner

**Affiliations:** ^1^Department of Psychology, York University, Toronto, ON, Canada; ^2^School of Psychology, The University of Queensland, Brisbane, QLD, Australia; ^3^Department of Psychology, University of California, Berkeley, Berkeley, CA, United States

**Keywords:** compassion, neuropsychology of compassion, genetics of compassion, trait stability of compassion, cooperation and compassion, theoretical models of compassion

What a pleasure to bring together this collection of articles from renowned researchers for this series on *The Expanding Science of Compassion*. We showcase research from neuroscience, epidemiology, experimental, developmental and social psychology. We also present novel contributions from the field of epi-genetics and meditative practices associated with compassionate behaviors. This collection helps illuminate the biopsychosocial angles from which we can understand compassion, and offers solutions toward a more compassionate world.

Ho et al. bridge three separate literatures that converge on the intra and interpersonal dynamics necessary for the transmutation of conflict into enduring peace. The path of “intuitive compassion” draws on a capacity to overcome the zero-sum mentalities, tit-for-tat strategies, and invalid beliefs that lead to afflictions, both on the personal and collective levels. Mahayana Buddhism over the last 2,000 years has emphasized the ultimate wisdom in merging personal and collective goals and transmuting conflict for the benefit of all sentient beings. In the prisoner's dilemma, the players of the game must also adopt a non-zero sum mindset to achieve optimal payoff over reiterated rounds. This strategy also referred to as “tit-for-tat with forgiveness” demonstrates how cooperation must be initiated and reciprocated for the beneficial outcome of both players. Processes within close relationships also illustrate how an atruistic and cooperative mindset reduces stress and benefits the individual. This literature is particularly relevant in recent geopolitical disruptions where “tit-for-tat” strategies have spiraled into ever increasing cycles of destruction. How could forgiveneness ever be introduced in such group conflicts?

Kirkland et al. use an experimental design to show that a pro- sharing group norm can inspire its members to behave more cooperatively toward other groups that have fewer resources. The research suggests that group dynamics and organizational culture can be influenced through the example of single members who demonstrate pro-sharing attitudes. This study found that the intervention involving a sharing model was more powerful than the condition involving the practice of compassion meditation at the individual level. Groups often fail to see the superordinate goal aiming at the benefit of all and superseding individual interests, particularly in contexts of inequality. This work has tremendous implications for the global community to orient groups dynamics toward the greater good through concrete examples in action.

Callaghan et al. show that we are less likely to give to members of lower socioeconomic standing compared to those within our own social standing. In their field experiment, the authors manipulated social status symbols worn by a confederate requesting money from pedestrians. Confederates wearing lower-class symbols were perceived more negatively and given less money than to those wearing symbols of higher social standing. The results remind us that we are more likely to be compassionate and generous toward those who are more “like us.” Ironically, it is those who need the most help who may be judged as less deserving.

Addiss et al. provide one of the first epidemiological review including 82 studies to identify individual and situational factors quantitatively associated with compassion. Their findings indicate that individual demographic factors related to compassion include being female and being spiritual or religious. In the area of personal dispositions and skills, empathic concern was strongly related to compassionate responding as well as secure attachment. A strong association was also reported between eudaimonia, prosocial personality traits and compassion. Compassion was more likely to occur in domestic settings and more likely to be expressed toward close ones. On an organizational level, ethical and compassionate leadership was found to relate to compassion among employees. In fact, the perception of one's organizational unit as being fair and compassionate was associated with self-reported levels of compassion. The patterns emerging from this epidemiological study emphasize that organizational culture, and commitment to ethical principles that can help nurture compassion among the collective workforce.

Further evidence for the contribution of traits toward prosociality comes from Paz et al. who report a moderately stable disposition to help from toddlerhood to early childhood. They demonstrate individual differences that appear early (18 months!) in the spontaneous demonstration of concern for others' welfare. Prosociality was assessed through various experimental tasks that elicited helping, sharing, and comforting behaviors. These different aspects of prosocial behaviors were found to be inter-related and fairly stable in early childhood, demonstrating trait-like characteristics.

There are many reasons to prioritize compassionate responding, regardless of its moral or practical necessity. For one, Dobewall et al. using telomere biology report a connection between prosocial traits and longevity. The data was obtained from the Young Finns Study examining six birth cohorts from 1997 to 2011. The authors found significant links between helpfulness, cooperativeness, and compassion toward others with less accelerated aging. This requires replication as not all epigentic indicators were significantly related to compassion. Nonetheless, research into the biological pathways linking prosocial traits to longevity are both intriguing and worth pursuing.

Perhaps one reason why helping others may bring a long life has to do with sleep. Witvliet et al. tested interventions administered right before sleep that were designed to either cultivate compassion or stimulate rumination toward a perceived offender. Compared to the rumination condition, those who engaged in compassionate reappraisal of the offender in a personal offense fell asleep faster and had fewer sleep disturbances. The authors recommend this intervention to promote empathy and forgiveness, as well as calmer and more restorative physiological states.

Fraser et al. report that relational compassion among newly weds may also entail better sex. Their results show that women's and men's compassionate behaviors (including mindfulness, engagement, forgiveness and gratitude) contributed to their partner's sexual wellbeing over time. This is important given the role of sexual satisfaction and intimacy for the strengthening of relationships particularly in the early stages of marriage.

Gilbert et al. show that compassion training can enhance an individual's willingness to engage with the suffering of others' and increase compassion toward oneself as well. They report on a new compassion technique, using visualization and music to engage meditatively with the suffering in the world. After 2 weeks of regular practice, participants reported significant increases in compassion toward others as well as toward themselves. This study helps to broaden our understanding of the mechanisms that stimulate growth in individuals' compassionate skills and brings hope that this capacity for human goodness can be enhanced.

Shubair et al. remind us that self-care is necessary and integral to the provision of compassion. They employed qualitative methods to identify the factors necessary to maintain optimism and engagement while avoiding compassion fatigue. The sample was drawn from social work instructors in higher education who were interviewed in depth. Themes emerging from the qualitative analyses indicated the necessity for balance, appropriate boundaries, and self-care to maintain compassion and joyfully engage in a caring way.

Rodríguez-Nieto et al. add to the existing literature on the neural biological substrates of the cognitive and affective components of compassion. Their fMRI data replicate previous brain imaging studies on the brain regions associated with compassion. More interestingly, they report gender differences in the connectivity of brain areas corresponding to the cognitive and affective components of compassionate responding. Despite the extensive overlap in the brain areas activated, there were also distinct neurocognitive pathways for men and women in response to the compassion stimuli. The authors suggest that different routes may be employed by each gender to arrive at similar compassionate responses. The authors also note that a larger sample size is necessary to replicate these effects. However, this study represents an important starting point for future work on potential gender differences in the recruitment of cognitive vs. affective components underlying compassionate responding.

In conclusion, this Research Topic includes some of the latest advances in the research on compassion, using a wider range of methods to illustrate the genetic, neural, interpersonal, societal, and individual underpinings of this cardinal human ability. Facilitative conditions as well as situational hinderances in the expression of compassion are showcased. The papers in this Research Topic are generally consistent with the possibility that compassion can evolve through intentional effort and can serve to shape our culture and our future. We are facing challenges that will necessitate cooperative action over and above self-interest, and our collective task is clear. We need to prioritize compassion in all our human affairs in order to rejuventate and inject meaning into our lives and our most important goals. This series will hopefully inspire new research and produce applications that inform our progress toward a kinder world.

## Author contributions

MM: Writing—original draft, Writing—review & editing. JK: Review & editing. DK: Review & editing.

